# 1592. SHEA Featured Oral Abstract: Reducing Unnecessary Antibiotic Treatment for Asymptomatic Bacteriuria: A Statewide Collaborative Quality Initiative

**DOI:** 10.1093/ofid/ofac492.114

**Published:** 2022-12-15

**Authors:** Valerie Vaughn, Ashwin Gupta, Lindsay A Petty, Anurag N Malani, Danielle Osterholzer, Payal K Patel, Mariam Younas, Steven Bernstein, David Ratz, Elizabeth McLaughlin, Tawny Czilok, Jennifer Horowitz, Scott A Flanders, Tejal N Gandhi

**Affiliations:** University of Utah School of Medicine, Salt Lake City, UT; Michigan Medicine, Ann Arbor, Michigan; University of Michigan, Ann Arbor, Michigan; Trinity Health St. Joseph Mercy Ann Arbor, Ann Arbor, MI; Hurley Medical Center/Michigan State University College of Human Medicine, Flint, Michigan; Intermountain Healthcare, Ann Arbor, MI; Hurley Medical Center/ Michigan State University College of Human Medicine, Flint, Michigan; Michigan Medicine, Ann Arbor, Michigan; VA Ann Arbor Health System, Ann Arbor, Michigan; Michigan Medicine, Ann Arbor, Michigan; Michigan Medicine, Ann Arbor, Michigan; Michigan Medicine, Ann Arbor, Michigan; University of Michigan/Michigan Medicine, Ann Arbor, Michigan; Michigan Medicine, Ann Arbor, Michigan

## Abstract

**Background:**

Up to one-third of hospitalized patients treated for urinary tract infection (UTI) have asymptomatic bacteriuria (ASB). Both diagnostic (avoiding inappropriate urine cultures) and antibiotic stewardship (reducing unnecessary antibiotic use in asymptomatic patients) have been proposed to reduce unnecessary antibiotic use for ASB. However, it's unclear which method is most effective.

**Methods:**

The Michigan Hospital Medicine Safety Consortium aimed to improve antibiotic use and outcomes of hospitalized patients with a positive urine culture between 7/1/2017—3/31/2020 (see **Table 1** for patient characteristics) by benchmarking performance across 46 Michigan hospitals, sharing best practices, and implementing pay-for-performance metrics related to unnecessary treatment of ASB (see **Figure 1**). Using logistic regression models controlling for hospital clustering, we assessed change over time in percentage of hospitalized patients treated for UTI who had ASB (i.e., had no documented signs or symptoms of UTI) and hospital characteristics associated with baseline or change in unnecessary antibiotic prescribing. We then estimated the percentage of avoided ASB treatment attributable to diagnostic (decrease in urine cultures ordered on asymptomatic patients) vs. antibiotic stewardship (decrease duration or avoidance of antibiotic treatment).

**Results:**

Across 46 hospitals, there were 15,493 patients with a positive urine culture. Of 13,805 patients treated for a UTI, 23.2% (3,197) had ASB. The percentage of patients treated for UTI who had ASB declined over time from 29.0% (95% CI: 26.1%, 32.0%) to 16.9% (95% CI: 14.2%, 20.1%; aOR 0.94 per quarter, 95% CI: 0.92-0.96; **Figure 1**) with hospitals not belonging to a larger healthcare system having the largest decrease over time (**Table 2**). Neither the proportion of patients with ASB who were treated with antibiotics (P=0.07) nor the duration of therapy for ASB changed over time (P=0.09); thus, nearly all avoided antibiotic therapy for ASB appeared due to diagnostic stewardship.

**Conclusions:**

Across 46 hospitals, there was a decrease over time in unnecessary treatment for ASB with independent hospitals improving most. Diagnostic stewardship appeared responsible for nearly all improvement.

**Disclosures:**

**Payal K. Patel, MD, MPH**, Qiagen: Honoraria.

